# Aging, cancer, and cancer vaccines

**DOI:** 10.1186/1742-4933-9-4

**Published:** 2012-04-17

**Authors:** Paolo Mazzola, Saba Radhi, Leonardo Mirandola, Giorgio Annoni, Marjorie Jenkins, Everardo Cobos, Maurizio Chiriva-Internati

**Affiliations:** 1 Department of Clinical and Preventive Medicine, University of Milano-Bicocca, Geriatric Clinic, San Gerardo University Hospital, Monza, Italy; 2 Department of Internal Medicine, Division of Hematology/Oncology, Texas Tech University Health Sciences Center, 3601 4th St, Lubbock, TX 79430, USA; 3 Department of Medicine, Surgery and Dentistry, Università degli Studi di Milano, Milan, Italy; 4 The Laura W. Bush Institute for Women's Health and Center for Women's Health and Gender-Based Medicine, Texas Tech University Health Sciences Center, Amarillo, TX, USA

## Abstract

World population has experienced continuous growth since 1400 A.D. Current projections show a continued increase - but a steady decline in the population growth rate - with the number expected to reach between 8 and 10.5 billion people within 40 years. The elderly population is rapidly rising: in 1950 there were 205 million people aged 60 or older, while in 2000 there were 606 million. By 2050, the global population aged 60 or over is projected to expand by more than three times, reaching nearly 2 billion people [1]. Most cancers are age-related diseases: in the US, 50% of all malignancies occur in people aged 65-95. 60% of all cancers are expected to be diagnosed in elderly patients by 2020 [2]. Further, cancer-related mortality increases with age: 70% of all malignancy-related deaths are registered in people aged 65 years or older [3]. Here we introduce the microscopic aspects of aging, the pro-inflammatory phenotype of the elderly, and the changes related to immunosenescence. Then we deal with cancer disease and its development, the difficulty of treatment administration in the geriatric population, and the importance of a comprehensive geriatric assessment. Finally, we aim to analyze the complex interactions of aging with cancer and cancer vaccinology, and the importance of this last approach as a complementary therapy to different levels of prevention and treatment. Cancer vaccines, in fact, should at present be recommended in association to a stronger cancer prevention and conventional therapies (surgery, chemotherapy, radiation therapy), both for curative and palliative intent, in order to reduce morbidity and mortality associated to cancer progression.

## Introduction

Elderly patients represent a unique and challenging group of patients to the practicing oncologist. They represent a heterogeneous group in terms of comorbidities and functional status which makes it difficult to establish management recommendation. One of the cancer pathways that is of interest in the elderly is the immune system and its role in oncogenesis and as potential therapeutic targets. In this review we present an overview of the changes in the immune system and the use of cancer vaccines in the elderly. We will also discuss the assessment of elderly patient with cancer.

## Aging and immunosenescence

Aging is a process characterized by progressive functional decrease in all organs, morphological changes, and immune system-related changes at the cellular and molecular levels, which determine less adaptive biologic functions. The immune system alterations in the elderly are comprehensively known as immunosenescence [1]. This phenomenon is characterized by an accumulation of changes that progressively results in dysfunctional or compromised immune responses [2,3]. Multiple aspects are involved: thymic involution [1], shifts in the number, distribution, and activity of T- [4] and B-lymphocytes [5-10], reduced availability of naïve CD4^+ ^and CD8^+ ^T-cells [1] and impaired production of naïve B-cells in bone marrow [11-13], dysfunction of antigen presenting cells (APCs) [3], alterations in cytokines production [9-13], frequent oligoclonal and monoclonal immunoglobulin production [10-23], skewing of B cell production to CD5^+ ^B cells that are more likely to generate auto-antibodies [11-15].

In detail, overall diversity of the total T-cell repertoire is skewed by oligoclonal expansions of memory CD4^+^, CD8^+^[10-23] and CD95^+ ^T-cells, and a limited production of naïve cells. Additionally, the increased memory and activated effector CD8^+ ^T-cells [24,25] show a restricted TCR repertoire diversity, have shorter telomeres [26] and a limited proliferative potential [27]. They are largely represented by clonally expanded populations reactive towards cytomegalovirus (CMV) and Epstein-Barr virus (EBV) determinants [24]. Such expansion significantly reduces T-cells available for responses against other infections or cancer. Although the thymus remains in part functionally competent [2], the diminished export rate associated with aging is insufficient to replace peripheral naïve T-cells lost.

There is also evidence of an increased concentration of IL-6, TNF-α, and various acute-phase proteins, suggesting that aging is associated with low-grade inflammatory phenotype [10-13,28,29], despite the absence of any particular disease [30,31].

Consequently, due to these alterations in the adaptive immune function, the elderly shows increased sensitivity to infectious diseases and cancer, and poor responses to vaccination [32,33]. Different studies have proven also that cancer vaccines are less effective in older individuals than in young adults [3,10-23,34,35].

## The chronic antigenic stress theory

Naïve T-cells able to specifically recognize a particular antigen are usually very few. In order to efficiently respond to an antigenic stimulus, they are able to rapidly perform many cell divisions, producing multiple clones. Once the acute antigenic challenge is resolved, excess clones undergo apoptosis, and the organism retains a certain number of memory cells [34]. If the exposure of T-cells is prolonged, the antigenic stimulation could become chronic, potentially contributing to a pro-inflammatory phenotype [35] and determining persistent T-cell clonal expansion. This scenario is commonly observed in the case of cancer (tumor-associated antigens), autoimmunity, and during the aging process (prevalently due to chronic stimulation by CMV antigens) [28,36]. The accumulated clones, whose number (absolute number and number of expanded cell lines) represents an important component to determine the immune risk profile of an individual [28,29], physically occupy a part of the "immunological space" [3] and probably alternate/suppress the immune responses of other specific clones [2]. Moreover, CMV-reactive clones are dysfunctional: they present the characteristics of anergic cells and a marked apoptosis resistance [32,33]. A study from Mazzatti DJ et al. has also demonstrated that chronic antigenic stress leads to gene expression changes in cultured T-cell clones [37]. Researchers have initially paid particular attention to the CD8+ anti-viral effectors, but also the CD4+ T-helper arm of the immune response seems to suffer the consequences of chronic CMV stimulation [38]. Whether this chronic exposition to a number of antigens (CMV, EBV [39], HIV [40], HCV [41], tumor antigens such as in melanoma [42,43]) has a role in driving immunosenescence needs to be further investigated, but the presence of expanded dysfunctional T-cell clones and the comprehension of this phenomenon probably represents a key factor for the development of new strategies of immune intervention in the elderly.

## Cancer immunoediting

### Cancer Immunoediting

is a term coined by Schreiber and colleagues to describe a process originating from the interaction between the host immune system and tumor cells [6-23].

Although aimed to prevent auto-immunity, tolerance can be directed towards non-self antigens, following a process called *induced tolerance*. Cancer originates from the transformation of the host cells: during this process, multiple accumulating mutations turn the "self" into a "non-self". This transformation is expected to trigger an immune response, but tumor immunity is different because of a number of possible mechanisms of immune evasion. The cancer cells often display weak immunogenicity, especially due to the lack or low expression of co-stimulatory molecules (such as B7) or inefficient antigen presentation ability. Therefore, potentially tumor-reactive T-cells could be induced to mount ineffective immune responses or even to present anergy.

At advanced stages of carcinogenesis, the immune system exerts a selective pressure on the genetically unstable tumor cells: those able to resist to or suppress the immune response are selected. This phenomenon, known as *immunoselection*, represents the first cause of immune escape.

Later in the process of tumor progression, inefficient immune responses can even favor tumor growth, in a process known as *immunosubversion *[39].

This complex sequence of events is known as *cancer immunoediting*: effective recognition corresponds to the *elimination *of transformed cells, but tumor cell variants can survive this process and enter an *equilibrium *phase, followed by an uncontrolled tumor expansion termed *escape*.

It is evident that elimination (firstly described as "immune surveillance hypothesis" by Burnet M. in 1957 [44] represents a critical factor controlling carcinogenesis, and that immunosenescence plays an essential role in promoting cancer immunoediting.

## Cellular senescence

Combined mechanisms are responsible of cellular senescence in vivo. It has been hypothesized that aging could be the result of the progressive addition of molecular damages, or that could be genetically pre-determined.

A number of evidences support the pre-determination of cellular senescence. One of the most relevant factors affecting cell aging is telomere shortening through multiple cell cycles. Accordingly telomerase, a specific enzyme preserving telomeres length, is poorly expressed in most human non-dividing cells and its levels decline with cell aging. Because telomeres protect chromosomal DNA from damages activating programmed cell death pathways (apoptosis), their shortening is thought to function as a "sensor" of cells age [13-23,40]. Perhaps not surprisingly, tumor cells abnormally express telomerase, which allows the maintenance of telomeres' length even after repeated replications. This potentially results in tumor "immortality". Other cellular senescence-related mechanisms are telomere-independent. Environmental modifications (e.g. increments of oxygen concentration and free oxygen radicals, DNA damage) can induce the aging process independently from telomere length [41-43].

In conclusion, cellular senescence is a genetically determined process that can also be induced by various alterations in the tumor environment. Studies on advanced cancer disease have rarely demonstrated the presence of senescent cellular phenotypes [45,46]. Furthermore, inflammation has also been shown as a promoter cellular senescence. Still too many interactions remain to be clarified, considering that the accelerated aging phenotype has also been evidenced in other diseases, e.g. in atherosclerosis, COPD [47] and liver fibrosis [48].

## Inflammaging

Not only adaptive responses, but also innate immune system is dysregulated during the senescence. The term "inflammaging" has been coined in 2000 by Franceschi C. et al. [35] to describe the phenomenon, triggered by the unspecific innate immunity, of chronic low-grade systemic inflammation which accompanies the aging process. Moreover, this condition represents a "common soil" for different age-associated chronic diseases, such as diabetes, atherosclerosis, Alzheimer's disease [32,35], arthrosis/arthritis, cardiovascular diseases, and cancer [32].

**Figure 1:**
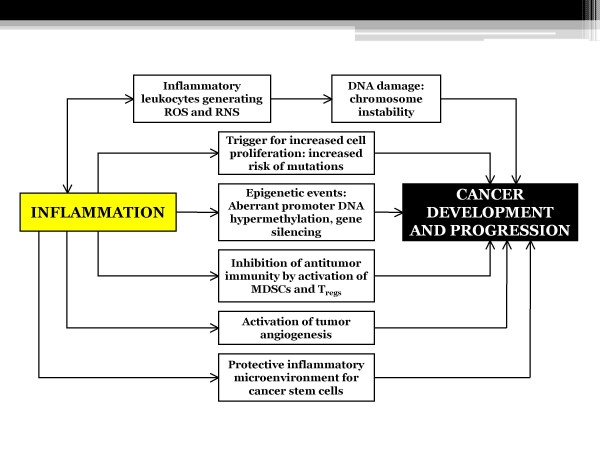
**Pro-inflammatory mechanisms probably involved in cancer development and progression at old age**.

The association between chronic inflammation and cancer has been widely described in different organs: inflammatory bowel diseases (IBDs) determine an increased incidence of colorectal cancer, while chronic gastritis is associated with gastric adenocarcinoma, and chronic viral hepatitis often leads to liver cancer. Various mechanisms account for inflammation-related carcinogenesis (Figure 1).

1. inflammatory leukocytes generate reactive oxygen and nitrogen species (ROS and RNS, respectively), causing local tissue alterations and DNA damage

2. enhanced proliferative signals mediated by cytokines released by inflammatory cells increase the risk of mutations

3. alterations in epigenetic mechanisms alter gene expression patterns

4. inflammatory mediators affect suppressor cell populations, particularly MDSCs and T-regulatory (Treg) cells, able to inhibit CD4^+ ^and CD8^+ ^T-cell proliferation, to block NK cell activation, to limit DC maturation, and to polarize immunity towards a type-2 helper response

5. the inflammatory microenvironment favors tumor-associated angiogenesis

6. cytokines generate a preferential niche supporting cancer stem cells (e.g. IL-4 for colon carcinoma stem cells).

In this scenario, where a number of alterations involve multiple cellular mechanisms, there is a growing need to identify one or a few target molecules shared by all cancer cells, in order to synthesize new and more efficient treatments. One of the mechanisms that could be targeted for this purpose is represented by telomere shortening/elongation.

## The importance of telomerase

Telomeres maintain chromosomal stability through repeated cellular divisions. Their repeated sequence of non-coding DNA (TTAGGG) has the crucial function to preserve genomic information during cell replication, but it results in progressive telomere shortening. Once the limiting length is reached, signaling of chromosomal instability triggers cellular senescence and apoptosis, unless the cell has the ability to preserve telomere length.

The enzyme *telomerase *is a reverse transcriptase responsible for the maintenance of telomeres length. Since telomerase discovery in 1984 by C.W. Greider and E. Blackburn, it represented an attractive therapeutic target because of its role in aging and cancer. Telomerase has recently gained attention for its potential applications in cancer therapy, anti-aging research and treatment of chronic diseases.

Telomerase expression makes cells potentially "immortal", including *cancer stem cells*. Accordingly, telomerase has been found in a variety of human cancers (80-90%), a condition that satisfies the need for a tumor specific target and could define this enzyme as a hallmark of cancer disease because telomerase activity also is poorly or non-expressed in normal somatic cells.

## Telomerase-based therapies

Different approaches have been tailored to telomerase synthesis or activity.

1. Direct enzyme inhibition, through antagonists of different components of the macromolecular complex (such as hTERT or TERC)

2. Active immunotherapy, aiming to stimulate immune responses against tumor cells expressing hTERT. For instance, Antigen-Presenting Cells (APCs) displaying hTERT epitopes, are able to stimulate specific T-cells to react towards telomerase-expressing tumors, also activating hTERT-specific memory T-cells

3. Telomere disruption/alteration: blocking or altering telomere structure (e.g. by using mutant engineered forms of hTERC) may quickly induce a DNA damage response and consequent apoptosis. However, this approach could target telomeres in normal cells or G-rich chromosome regions, resulting in potentially lethal toxicities

4. Delivery of telomerase promoter-controlled suicide genes, able to induce apoptosis selectively in telomerase-positive cells

5. Blocking telomerase expression or functions would result in reduced enzyme activity in tumor cells. The potential disadvantage of these approaches is the need for prolonged therapies to maintain a clinical response.

Examples of drugs recently under clinical trials are GRN163, GV1001 and GRNVAC1. The first, from *Geron Corporation*™ http://www.geron.com, belongs to the class of direct telomerase inhibitors blocking the hTERC. GRN163 intracellular distribution has been recently improved by lipid conjugation [49] (GRN163→GRN163L).

GV1001 (GemVax/Pharmexa™) and GRNVAC1 (Geron Corporation™) are two active immunotherapy options, both able to elicit CD8^+ ^and CD4^+ ^T-cell responses. GV1001 is a hTERT peptide activating CD8^+ ^T cells (through MHC class I). GRNVAC1 consists in autologous DCs transduced with TERT-LAMP encoding mRNA. LAMP (*lysosomal-associated membrane protein*) favours lysosomal processing and presentation on MHC class II).

## Vaccine strategies in the elderly

Adoptive vaccination seems the most promising strategy to improve cancer vaccines in the geriatric population. It consists of transferring immunity through the administration of specific antibodies or immune cells, such as T-cells [50-52] or DCs [53]. DCs are the most relevant cellular determinants of vaccine-induced immunity through adaptive responses, but DC-mediated reaction is linked with innate immunity, since the induced antibody response activates the complement cascade [5-23,44,54-57].

However, even DC populations undergo important changes with advancing age.

## Aging and dendritic cells

Lots of studies have been conducted on animal models and in humans, demonstrating the decrease of DCs in number, distribution, and potentially, their generation and development from hematopoietic precursors [5-23,44,53,55-57]. Other observations showed that human thymic DCs decline in number (because of reduced thymic cellularity) but not in proportion in respect to young individuals [50-53]. Epidermal Langerhans Cells (LC), a skin and conjunctival subtype of DCs, also decrease in number with age and UV exposure, playing a permissive role in the development of skin cancers in the aged or sun-damaged skin [5-23,44,50-58]. In human blood, peripheral DCs are identified in two subsets: DC1, expressing CD11c and called "myeloid" (mDC), and DC2, expressing CD123 and called "plasmacytoid" (pDC). Teig N. et al. [53] revealed that CD11c + DC1 subpopulation did not change in the elderly, while CD123+ DC2 decreased.

In vitro studies found that DCs from young and old subjects expressed the same molecular patterns on their surface. Furthermore, DCs generated in vitro from elderly donors survived better under culture conditions. DCs from different healthy individuals were also equally effective with age in stimulating T cell responsiveness in vitro [50-54,58]. However, in vivo studies showed that DCs in elderly individuals are less able to stimulate immune responses. Finally, a comparison with DCs generated ex vivo from precursor cells evidenced their complete functionality; it seems that DC precursors are still able to differentiate into functionally active DCs in the elderly, with an appropriate stimulation. This was an evident and attractive potential target for therapeutic approaches in age-associated malfunctions of the immune system [5-23,44,50-58]. The problem is that unhealthy elderly patients, e.g. with cancer disease, may present with multiple impairments, both in T-cell responses and in APCs functions [5-23,44,50-58]. Further investigations are necessary to clarify the activation and differentiation signaling, to improve DC functionality when altered, and increase vaccine efficacy in the elderly.

## DC-based vaccines

In order to stimulate tumor-specific CTLs [58], DC vaccines may be performed by using:

1. whole tumor lysates

2. viral, bacterial or yeast vectors

3. specific proteins/peptides

4. nucleic acids

Autologous or allogenic tumor cells can be modified and administered in association with adjuvants or as tumor-cell lysates. The advantage of this method is the possibility that multiple and still unknown antigens can be simultaneously targeted. Moreover, whole tumor cell vaccines elicit MHC-I and -II responses. On the contrary, unfavorable aspects include a probability that unknown weak TAAs may trigger auto-immune responses.

Vectors are the more immunogenic way to deliver recombinant genes into DCs, which are better than direct administration with adjuvants. Issues include the choice of an efficient vector, and a balance between the stimulation of innate versus adaptive responses.

Proteins can be administered as single agents, in combinations, or as fusion proteins, and also peptides as single agents, agonist peptides, and anti-idiotype antibodies.

Advantages include cost-effective production, storage, and distribution. The identification and administration of TSAs is more accurate, leading to a low risk of inducing auto-immunity respect to tumor cells. On the other hand, single proteins or peptides are weakly immunogenic, and tumor antigen mutations or loss can easily escape immune recognition. Another disadvantage is represented by HLA-restricted responses that limit their use to selected patients. Finally, their poor ability to induce balanced activation of CD4^+ ^CD8^+ ^subsets leads to a less effective long-term anti-tumor immunity.

The consecutive administration of different TSAs, in different time points, has shown better outcomes than the simultaneous one.

DNA-based vaccines are a strategy capable of activating strong immunity against weak TAAs. Approaches to enhance their potency include improved delivery systems, co-administration of cytokines, or the use of separate plasmids encoding non-self antigens; mRNA vaccines are based on transient transfection of non-dividing cells: the transfected mRNA does not integrate into the host genome [59], determining an high grade of safety. Further transfection efficiency may be obtained by a procedure called electroporation [60]. The rationale of these vaccines is the translation into protein of mRNA-coding TAA transfected into DCs; then, after protein processing, the synthesized antigen is loaded on MHC molecules for antigen presentation, activating an antigen-specific CTL-response [61,74-77].

However, triggering the immune response alone seems to be an incomplete strategy of vaccination because of multiple cited factors harming the immune system.

## Assessment of elderly patients with cancer

The heterogeneity of the elderly makes managing the older patient with cancer very challenging. Chronological age does not correlate with better response or increased toxicity to treatment. There are no known criteria to help decide management plan as older adults are underrepresented in oncology clinical trials that set the standard of care. The Comprehensive Geriatric assessment (CGA) has been suggested as a tool to evaluate elderly patients with cancer. It includes multidimensional assessments, including comorbidities, functional status, cognition, psychological state, nutrition, social conditions and medications. This provides a detailed assessment of the patient which helps to individualize management. Malnutrition and functional statuses were found to independently predict change in cancer management especially in the vulnerable elderly [56].

## Cancer therapy in the elderly

The flow-chart highlights the different possibilities of treatment administration and association, focusing on cancer vaccination handiness, independently from disease staging and treatment purpose.

When the risk of morbidity and mortality from neoplastic disease is low considering life expectancy and severity of co-morbidities, the choice should be palliation, including:

• Management of co-morbidities

• Symptomatic approaches (control of pain and cancer-related symptoms)

• Supportive medical and psycho-social care.

**Figure 2:**
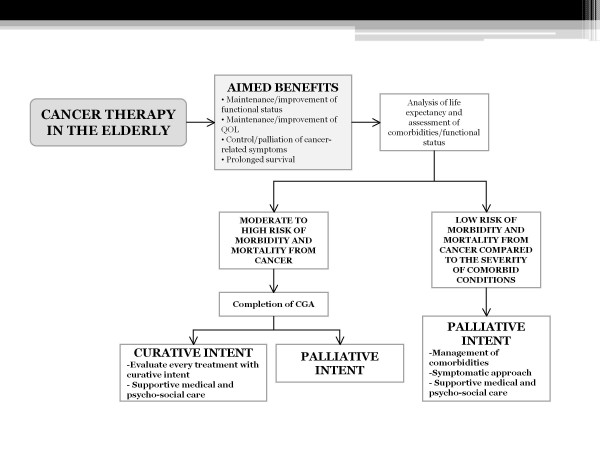
**The importance of estimating life expectancy and comorbidities, in association with the Comprehensive Geriatric Assessment, in order to guide cancer therapy in the elderly**. Independently from the intent, the maintenance of Quality of Life and the control of cancer-related symptoms should be the first aim of the treatment.

If the estimate of life expectancy and the assessment of co-morbidities and functional status determine a moderate to high risk of morbidity and mortality from cancer during the lifespan, then a complete clinical and psycho-social evaluation should be performed in order to estimate a realistic risk/benefit ratio (Figure 2).

**Figure 3:**
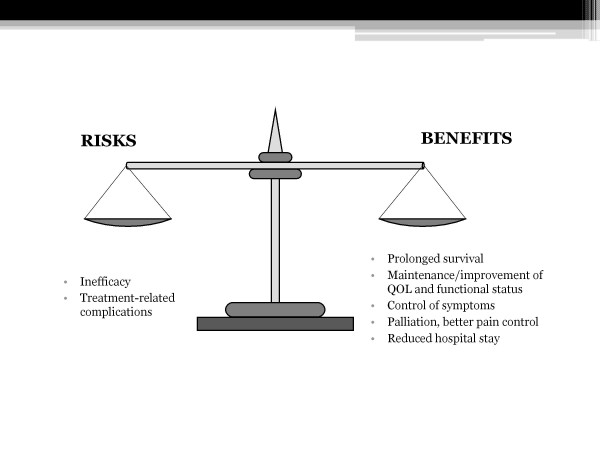
**Potential risks and benefits of cancer therapy in the elderly**.

Apart from the patient's underlying medical characteristics and the number of conditions affecting the immune system during cancer disease, the true risk factors for a cancer therapy are represented by its inefficacy and side effects (Figure 3).

**Figure 4:**
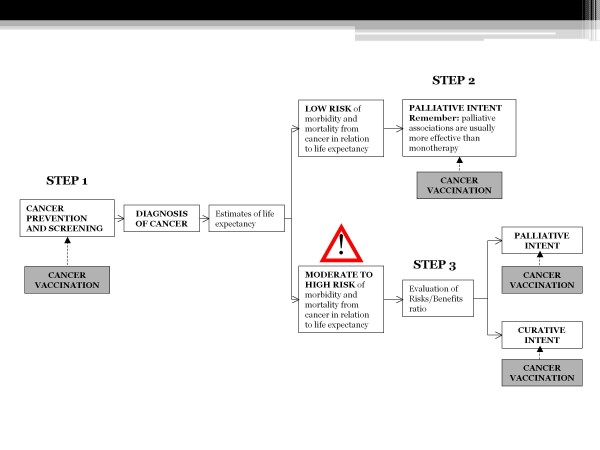
**Possible decisional algorithm for cancer therapy in the elderly, from screening to treatment, Step 1-2**.

**Figure 5:**
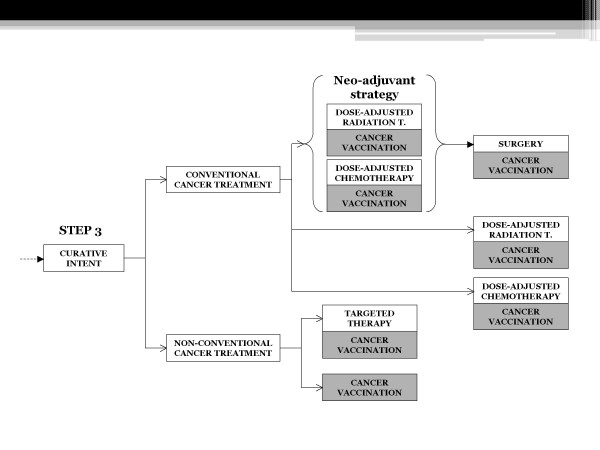
**Possible decisional algorithm for cancer therapy in the elderly, Step 3**. The entire flow-chart enlightens the possibilities of administration and association of cancer vaccines at different steps of the evaluation of malignancies, from prevention to curative and palliative treatments.

If risk/benefit ratio is acceptable, conventional curative options include surgery, radiation therapy and chemotherapy, usually dose-adjusted (Figures 4 and 5).

Surgical approaches may represent the first option for solid tumors; elective surgery in the elderly cancer patients, and in the geriatric population in general [62,73], have shown similar outcomes as in the younger ones [63]. This consideration warrants performing a CGA and preoperative assessment is required [64]. Geriatric surgery is about disease and functional status, not age. Early mobilization and rehabilitation after surgery were shown to reduce the incidence of post-operative complications. In association with this option, cancer vaccination could find application both in neo-adjuvant and adjuvant strategies, co-administered with Radiation Therapy (RT) or CT.

Both brachytherapy and external beam source can be proposed either in the curative or palliative setting. RT is usually effective and well tolerated. However standard protocols and/or doses sometimes need to be modified according to patient's conditions or to the onset of complications [65]. As for surgery, age per se does not represent a limiting factor to the prescription of RT [66,67].

Chemotherapy may be considered as a first-line approach, as well as an adjuvant in combination with RT, or as palliative treatment. In any case, it should always be individualized in the elderly. Clinical trials have focused mainly on young and adult populations, and in a few cases, on a selected, much healthier population of "young old" (65-75 years old) or "old" patients (76-85 years old). However, only a relatively small percentage of the geriatric population is actually fit and capable of tolerating standard dose CT regimens [68]. The increased incidence and prevalence of multiple co-morbidities, their severity, and the presence of functional impairment (measured by the means of ADL scale), act as potential additional risk factors or contraindications involving dose/schedule adjustments or drug substitution at baseline. Patients with poor functional statuses, decreased social activities, and poor hearing were found to be at increased risk of chemotherapy toxicity [57], Moreover, once CT is established, it is also affected by an high incidence of multiple side effects: hydration and electrolyte imbalances, gastrointestinal effects (nausea, vomiting, diarrhea) and consequences (malnutrition), infections (with or without leucopenia), functional decadence and dependence, delirium and/or accelerated cognitive dysfunction, myelosuppression (causing anemia, thrombocytopenia, neutropenia), mucositis, organ-specific toxicity (cardiac, renal), sense organ deficits (hearing-visual impairment), peripheral and/or central neurotoxicity. All these factors contribute to the eventual discontinuation of CT, adjusted doses or a change in the regimen used. A number of studies have been performed in order to decrease, prevent, and treat these conditions. Several recommendations produced in recent years resulted in a better control of CT-related complications, but these conditions remained serious problems, sometimes refractory to all kinds of treatments. Comprehensive supportive care (aggressive intravenous rehydration, nutritional and vitamin supplements, prophylactic hematopoietic growth factors [69-71], blood transfusions, iron therapy, organ-specific monitoring [72,73], hospitalization if necessary) is therefore crucial throughout the CT course.

"Targeted therapies" represent a recent therapeutic pharmacological option for a number of malignancies, and are based on the concept that molecular changes responsible for malignant transformation can be targeted by specific agents. The aim of these treatments is to discriminate cancer cells, reducing adverse effects on healthy cells and improving overall benefits. This option captured increasing interest in geriatric cancer patients' care, yet beneficial and adverse effects should be further analyzed in this segment of the population, also for those patients who are unable to tolerate cytoreductive CT. Once more, cancer vaccination could be associated with this option or also administered alone in this group of patients.

However, inclusion of the elderly (fit and unfit individuals) in cancer vaccine clinical trials is warranted, and a careful selection of patients who would benefit from this treatment is critical [5-23,44,55-57]. Biological drugs can also be used in association with RT, CT, or both, only after an accurate re-evaluation of the risk/benefit ratio because of the important reported side effects [37,55].

## Conclusion

Cancer prevention remains critical in the geriatric, as well as in the young population [5-23,44,55-57].

The development and use of cancer vaccines also should be widely encouraged [5-23,44,55-57]. The immune system in elderly cancer patients is weakened by age-related changes and usually by the immunosuppressive effects of conventional treatments: this condition may suggest the use of cancer vaccination approaches before or together with other treatments. In any case, to identify and understand which alterations occur in the senescent immune system is the only way for individualizing, optimising and enhancing the antitumor immune responses in the geriatric population. Although further and great improvements in cancer vaccines are warranted, at present this approach should be suggested in association with systematic cancer prevention and conventional therapies (surgery, chemotherapy, radiation therapy). When cancer is involved, both in the young and in the elderly population, the strategy should be: "treat early, treat often".

## Abbreviations

CGA: Comprehensive Geriatric Assessment; IBD: Inflammatory Bowel Disease; MDSC: Myeloid-Derived Suppressor Cell; QOL: quality of life; TAA: Tumor-Associated Antigen; TCR: T-Cell Receptor; TSA: Tumor-Specific Antigen; TNF: Tumor Necrosis Factor.

## Competing interests

The authors declare that they have no competing interests.

## Authors' contributions

PM, LM, SR, MCI and EC reviewed the current literature. PM wrote the manuscript and MCI, GA, SR, LM, EC, MJ revised the manuscript. MCI conceived and drafted the manuscript. MCI, EC, GA approved the final version.
